# Dependence of Heart Rate Variability Indices on the Mean Heart Rate in Women with Well-Controlled Type 2 Diabetes

**DOI:** 10.3390/jcm10194386

**Published:** 2021-09-25

**Authors:** Adriana Robles-Cabrera, José M. Torres-Arellano, Ruben Fossion, Claudia Lerma

**Affiliations:** 1 Biomedical Sciences, National Autonomous University of Mexico, Mexico City 04510, Mexico; adriana.robles@c3.unam.mx; 2 Department of Electromechanical Instrumentation, National Institute of Cardiology Ignacio Chávez, Mexico City 14080, Mexico; jose190288@live.com.mx; 3 Center for Complexity Sciences (C3), National Autonomous University of Mexico, Mexico City 04510, Mexico; ruben.fossion@nucleares.unam.mx; 4 Institute for Nuclear Science, National Autonomous University of Mexico, Mexico City 04510, Mexico

**Keywords:** type 2 diabetes mellitus, women, heart rate variability, mean heart rate, orthostatic challenge, rhythmic breathing

## Abstract

Heart rate variability (HRV) is a method used to evaluate the presence of cardiac autonomic neuropathy (CAN) because it is usually attributed to oscillations in cardiac autonomic nerve activity. Recent studies in other pathologies suggest that HRV indices are strongly related to mean heart rate, and this does not depend on autonomic activity only. This study aimed to evaluate the correlation between the mean heart rate and the HRV indices in women patients with well-controlled T2DM and a control group. HRV was evaluated in 19 T2DM women and 44 healthy women during basal supine position and two maneuvers: active standing and rhythmic breathing. Time-domain (SDNN, RMSSD, pNN20) and frequency-domain (LF, HF, LF/HF) indices were obtained. Our results show that meanNN, age, and the maneuvers are the main predictors of most HRV indices, while the diabetic condition was a predictor only for pNN20. Given the known reduced HRV in patients with T2DM, it is clinically important that much of the HRV indices are dependent on heart rate irrespective of the presence of T2DM. Moreover, the multiple regression analyses evidenced the multifactorial etiology of HRV.

## 1. Introduction

Type 2 diabetes mellitus (T2DM) is a metabolic disease characterized by hyperglycemia due to alterations in the production and action of the hormone insulin, which is related to neuropathy and cardiovascular comorbidities [1]. Worldwide, there are more women than men with T2DM, and women have a worse prognosis [2]. The sex dimorphism presented in T2DM is associated with gender-sensitive external (socio-economic and environmental) and internal (genetic, epigenetic, metabolic, hormonal, and behavioral) factors [3]. In 2019, T2DM caused the death of approximately 2.5 million women between 50 and 70 years, reducing life expectancy by around 7 years [4]. T2DM patients have an increase in the hazard ratio for cardiovascular mortality between 60–70 years, which is associated with the development of cardiac autonomic neuropathy or “CAN” [5].

CAN is one of the most prevalent diabetes comorbidities, related to important causes of cardiac death such as lethal arrhythmias, silent myocardial ischemia, and sudden cardiac death [6]. This type of dysautonomia is characterized by sympathovagal imbalance, in which sympathetic activity predominates over parasympathetic activity [7], inducing an increase in heart rate and blood pressure [8] at the systemic level, and generating alterations in the cardiac conduction system and myocardial contractility [9]. Since the 1990s, heart rate variability (HRV) has been a tool widely used to indirectly measure activity from the autonomous nervous system (ANS), both from the sympathetic and parasympathetic branches, through its effect on heart rate (HR) [10]. In recent years, some studies have found a close and direct dependence of HRV on average HR in healthy human subjects in rest [11,12] and during cardiovascular reflex testing (the Ewing battery) [13,14], animal models and isolated heart tissue [15]. These studies argue that HRV does not give additional information on the ANS beyond average HR. However, other studies have demonstrated that HRV has prognostic power on morbidity and mortality in patients with cardiovascular and other diseases that is independent of the average HR and therefore should provide valuable extra information on cardiac autonomic regulation [16,17,18,19,20]. Therefore, the relation between HRV and average HR is not well understood in healthy human subjects, and even less so in the presence of pathology.

Although HRV has been studied in many samples of diabetic patients [21,22,23,24,25,26], the dependence of HRV on the mean heart rate of diabetic patients has not been documented. This study aimed to evaluate the correlation between the mean heart rate and the HRV indices in women patients with well-controlled T2DM and a control group. Multivariate linear regression analyses were applied to corroborate the dependence with other predictor variables such as age and the presence of T2DM.

## 2. Materials and Methods

### 2.1. Study Protocol and Patients

**Figure 1:**
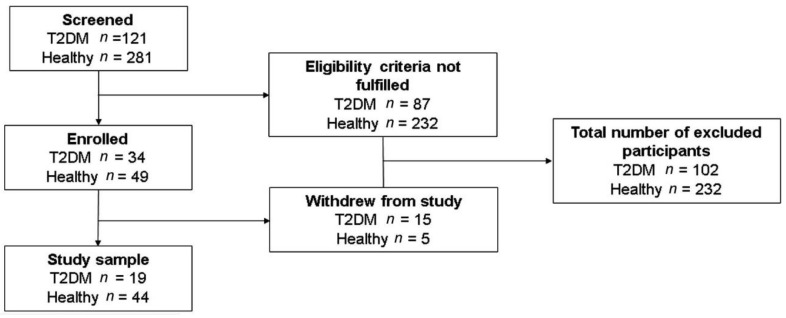
Diagram of enrollment including healthy controls and participants with type 2 diabetes (T2DM).

Diabetic patients (*n* = 19) were recruited from the Integral Care Center for Diabetic Patients (CAIPADI) of Instituto Nacional de Ciencias Médicas y Nutrición Salvador Zubirán (INCMNSZ). They were selected using strict inclusion criteria to ensure metabolic and blood pressure control (age 35–60 years, BMI < 30 kg/m^2^, HbA1c < 6.5%, fasting glucose < 130 mg/dL, LDL < 130 mg/dL, SBP < 140 mmHg, DBP < 90 mmHg, with no evidence of diabetic retinopathy or nephropathy diagnosed by a specialist, without any kind of acute or chronic diseases such as infectious, psychiatric, rheumatic, or gastrointestinal illness, among others, without antidepressant, anxiolytic or analgesic treatment). The healthy group (n = 44) were volunteers recruited from the Universidad Nacional Autónoma de México and the Instituto Nacional de Cardiología Ignacio Chávez, with the same inclusion criteria as the diabetic patients, except for fasting glucose <100 mg/dL. Subjects with electrocardiogram abnormalities, endocrine, cardiovascular, immune, or neurological diseases, or under hormonal treatment were excluded. The diagram of enrollment is shown in Figure 1.

From each participant a brief medical history was obtained according to the NOM-004-SSA3-2012 guideline. Anthropometric measures (height, weight, BMI, blood pressure, and waist circumference), and a blood sample (to obtain glucose, triglycerides, total cholesterol, HDL, LDL, creatinine, uric acid, and ureic nitrogen levels) were obtained. Finally, electrocardiogram recordings were obtained to assess HRV with the protocol described below.

### 2.2. Ethical Considerations

This study was conducted with the approval of the Ethics and Research Committee of INCMNSZ, registration number: CONBIOETICA-09-CEI-011-20160627, reference 3102, and the Institutional Ethics Committee of the Instituto Nacional de Cardiología Ignacio Chávez (protocol code 18-1090, approved on 30 October 2018). Each participant was asked to sign the informed consent and the privacy of their data was maintained. None of the participants obtained payment.

### 2.3. Materials

To measure weight and body mass index, an Omron HBF-514C scale was used. Blood pressure was measured with an Omron HEM-712C monitor and height with an InLab S50 portable stadiometer.

### 2.4. Blood Sample and Reagents

The blood samples were analyzed using spectrophotometry (filters 340–620 nm) with Advia 1800 Clinical Chemistry System (Siemens, Munich, Germany). Glucose-hexocinase_3 (GLUH_3, Advia Chemistry, Siemens, Munich, Germany), HbA1c 3M (Advia 1800, Siemens, Munich, Germany), uric acid (Uricase/Peroxidase, Advia 1800, Siemens, Munich, Germany), creatinine (alkaline picrate, Advia 1800, Siemens, Munich, Germany), triglycerides (GPO-PAP method, Advia 1800, Siemens, Munich, Germany), total-, LDL-, and HDL-cholesterol (catalase method, Advia 1800, Siemens, Munich, Germany).

### 2.5. Electrocardiogram Recording and Beat Detection

Electrocardiographic recordings were obtained with the Zephyr Bioharness device (Zephyr Performance Systems, Medtronic, Annapolis, MD, USA) using a sampling frequency of 250 Hz. 

The study was carried out between 7:00–9:00 a.m. at room temperature between 20–25 °C. The participants were under fasting conditions and it was requested that they not exercise or consume coffee, alcohol, or tobacco the day before. Subjects from the diabetic group were requested to take the medication after the study. The Zephyr Bioharness band was placed on the chest of the participants. The quality of the signal was verified in real time with the IoTool Platform Zephyr Sensors application (SenLab, Slovenia, Balkans). To start the recording protocol, each participant was asked to lie down on a stretcher, remain silent and with closed eyes. 

**Figure 2:**
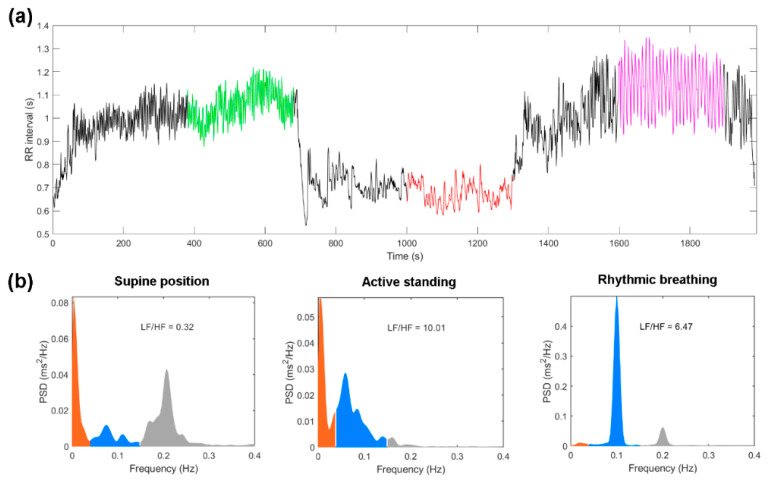
Example of HRV analysis from a healthy subject. (**a**) Time series obtained from the entire recording protocol with selected 5-min segments that we used in the HRV analysis are indicated with color: supine position (green), active standing (red), and rhythmic breathing (magenta). (**b**) Power spectrum density (PSD) from the selected segments: very low-frequency (orange), low-frequency (blue), high-frequency (grey), (LF/HF = ratio of low-frequency band power with respect to high frequency band power).

The HRV protocol was conducted under the considerations reported in Laborde et al., 2017 [27] to evaluate tonic (supine) and phasic HRV (active standing and rhythmic breathing), which shows the reactivity or response of the system to two different physiological challenges represented as changes in HRV (supine vs standing or supine vs breathing). The ECG was recorded in supine position (10 min), followed by two consecutive physiological maneuvers of active standing, and rhythmic breathing (at 0.1 Hz or 6 breaths per minute) of 10 min each (Figure 2).

Active standing is a hemodynamic stimulus in which the drop in blood pressure due to the gravitational force is avoided through an increased sympathetic activity towards the blood vessels (which increases peripheral vascular resistance) combined with a decreased parasympathetic activity (which increases heart rate) [28,29]. Rhythmic breathing is a stimulus that increases cardiorespiratory interactions through both the baroreceptors and the chemoreceptors [30]. While the cardiorespiratory interactions during spontaneous breathing usually influence HRV at around 0.25 Hz, rhythmic breathing at 0.1 Hz induces a strong cardiorespiratory coupling, and most of the power spectrum energy of the HRV shifts around this low frequency (0.1 Hz) [30].

Each ECG recording was transferred to a computer with the program BioHarness log downloader (Medtronic, USA) for further off-line processing and analysis. Identification of the QRS complex of each heartbeat was obtained with a computer program previously validated [31]. Miss-detected heartbeats were identified by visual inspection and corrected manually. The RR interval times series was measured from the period between consecutive QRS complexes. An adaptive filter was applied to the RR intervals to identify RR intervals from ectopic origin and to obtain RR intervals from sinus node origin only (NN intervals or HRV) [32].

### 2.6. HRV Analysis

The correct detection of the RR intervals was first evaluated in the software Processing [31] and the HRV parameters were measured in MATLAB R2020b. After the adaptation time (5 min), segments of 5 min were selected in supine position, active standing, and rhythmic breathing (Figure 2a).

For each time series, time-domain parameters such as average value of all RR intervals (MeanNN), standard deviation of all RR intervals (SDNN), square root of the average of the squared differences between adjacent R-R intervals (RMSSD), percentage of successive RR intervals with differences larger than 20 ms (pNN20) were calculated. To estimate the indices in the frequency domain, each time series was resampled with a linear interpolation of three samples per second and Welch’s periodogram method was used to estimate the density of the power spectrum. For the low frequency (LF) band (0.04 to 0.15 Hz) and high frequency (HF) band (0.15 to 0.4 Hz). The spectral power was calculated in normalized units (n.u). Time and frequency domain indices have been associated with autonomic activity. The HF index is reliably related to the control of vagal activity, the LF index reflects both parasympathetic and sympathetic activity [33]. Figure 2b shows a representative example of the power spectral density (PSD) in each analyzed segment.

### 2.7. Statistical Analysis

The statistical analysis was performed with the Statistical Package for Social Sciences (SPSS) version 25.0 (IBM, Armonk, NY, USA). A *p*-value < 0.05 was considered statistically significant. Normal distributions were confirmed in all study variables through a Kolmogorov–Smirnov test, except for LF/HF, which was transformed to logarithmic units. The results are described as mean ± standard deviation and were compared between groups by Student t-tests (two groups) or one-way ANOVA for repeated measures (three or more groups) with post-hoc analysis adjusted by the Bonferroni method. Bivariate correlations were calculated between the meanNN and each HRV index with Pearson’s method. Then, linear stepwise multiple regression analyses were performed with each HRV index with a dependent variable and meanNN, maneuver, diabetes mellitus (DM) condition, and age as independent variables. We used the stepwise format of the SPSS program, which “In each step, the independent variable that is not in the equation that has the lowest probability of F is entered, if that probability is small enough. Variables that are already in the regression equation are eliminated if its probability of F becomes large enough. The method ends when there are no more variables suitable for inclusion or elimination” according to the SPSS manual.

## 3. Results

### 3.1. Antropometric Measures

Table 1 shows the anthropometric characteristics of the study participants. Compared to the healthy group, the diabetic group was older, had smaller height, larger BMI, and a faster resting average HR. Both groups had similar weight, waist circumference, systolic blood pressure (SBP) and diastolic blood pressure (DBP).

### 3.2. Metabolic Profile

The metabolic profile variables were similar between both groups, except for higher serum glucose and uric acid values in the diabetic group compared to the healthy group (Table 2).

In our diabetes group (*n* = 19), normoglycemic treatment was used in 17 with metformin only, and 2 of them used a diet and exercise regime. The time since diabetes was diagnosed ranged between 0.8 to 10 years, with a median of 2 years. 

The participants who smoked totaled 14; 4 in the diabetes group and 10 in the healthy group. None of our participants were consumers of alcohol.

### 3.3. HRV Indices between Groups and Maneuvers

Table 3 shows the HRV indices during baseline and the two maneuvers (supine, active standing, and rhythmic breathing) in both groups. During supine position, the diabetic group had a shorter meanNN (i.e., faster heartbeat) and lower SDNN, RMSSD, and pNN20, indicating less variability in comparison with the healthy group. There were no differences between groups in the spectral indices during the supine position (LF, HF, LF/HF). During active standing, the diabetic group had lower SDNN, LF, and log LF/HF, and higher HF compared to the healthy group. During rhythmic breathing, the diabetic group had lower SDNN, RMSSD, and pNN20 compared to the healthy group.

Comparisons of supine position versus active standing within the healthy group showed the expected acceleration of heart rate (shorter meanNN), decreased variability (lower SDNN, RMSSD, pNN20), increased sympathetic activity (higher LF and log LF/HF) and decreased parasympathetic activity (lower HF). In contrast, the diabetes group responded to active standing with increased heart rate (shorter meanNN) and decreased variability (lower pNN20) but without significant changes in LF, HF, and log LF/HF. 

Rhythmic breathing induces more regularity and periodicity in the HR time series; in particular at the specific frequency of 0.1 Hz the amplitude of the oscillation is maximized, which translates into larger variability. In our study, the maneuver of rhythmic breathing induced similar responses in both groups: increasing variability and LF preponderance, because HR follows the same frequency as respiration and 0.1 Hz falls within the LF band.

### 3.4. Correlations between meanNN and HRV Indices

**Figure 3:**
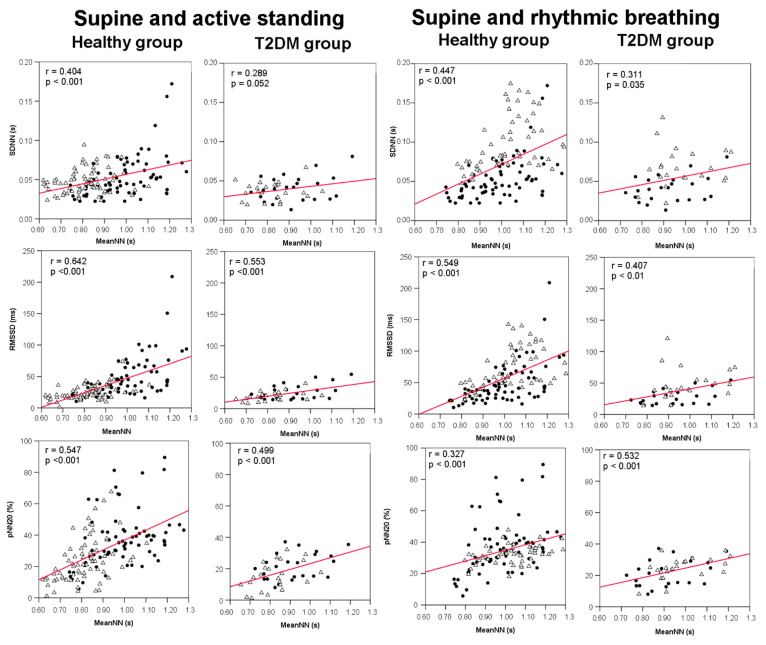
Dispersion plots between time domain HRV indices and meanNN in 44 healthy women (healthy group) and 19 female patients with type-2 diabetes mellitus (T2DM group). The plots on the left panels combine the HRV indices measured during both supine position (filled circles) and active standing (open triangles) of each group (*N* = 88 for the healthy group and *N* = 38 for the T2DM group). The plots on the right panels combine the HRV indices measured during both supine position (filled circles) and rhythmic breathing (open triangles) of each group (*N* = 88 for the healthy group and *N* = 38 for the T2DM group). MeanNN: mean value of all NN intervals; SDNN: standard deviation of all RR intervals; RMSSD: square root of the average of the squared differences between adjacent R-R intervals; pNN20: percentage of successive RR intervals with differences greater to 20 ms; r = Pearson’s correlation coefficient; *p* = *p* value.

**Figure 4:**
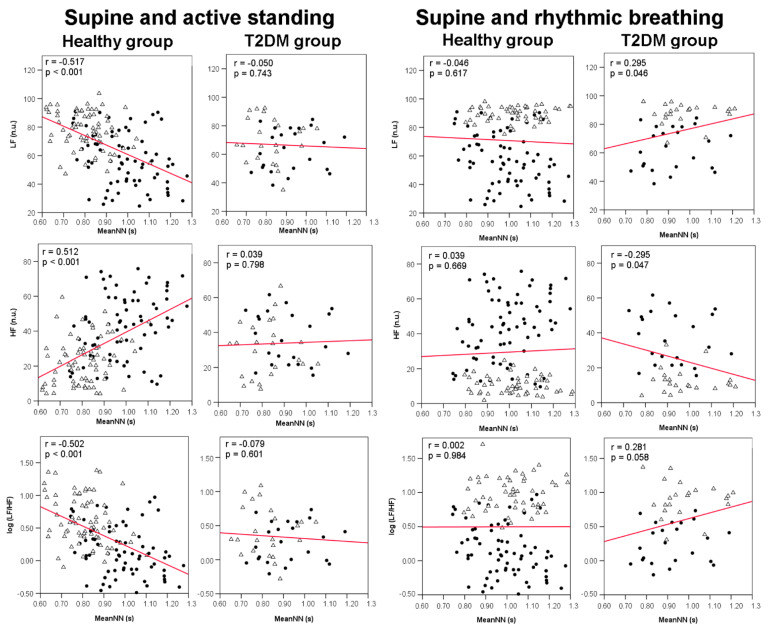
Dispersion plots between frequency domain HRV indices and meanNN in 44 healthy women (healthy group) and 19 female patients with type-2 diabetes mellitus (T2DM group). The plots on the left panels combine the HRV indices measured during both supine position (filled circles) and active standing (open triangles) of each group (*N* = 88 for the healthy group and *N* = 38 for the T2DM group). The plots on the right panels combine the HRV indices measured during both supine position (filled circles) and rhythmic breathing (open triangles) of each group (*N* = 88 for the healthy group and *N* = 38 for the T2DM group). MeanNN: mean value of all NN intervals; LF: low frequency band index; HF: high frequency band index; log (LF/HF): logarithm of the LF to HF ratio; n.u. = normalized units; r = Pearson’s correlation coefficient; *p* = *p* value.

The correlation analyses were applied combining the sample from the baseline condition (supine position) and each of the two maneuvers (either active standing or rhythmic breathing) to expand the range of meanNN variation (in the active standing maneuver) or HRV variation (in the rhythmic breathing maneuver). When we combined the data from the supine position and active standing to explore the bivariate correlations between each HRV index and meanNN (as independent variable), the healthy group showed the expected response to active standing in all HRV indices with significant correlations against meanNN (Figure 3 and Figure 4). In contrast, the diabetes group only showed significant correlations with RMSSD and pNN20 (Figure 3).

The combined data from supine position and rhythmic breathing showed that both groups have significant positive correlations in time-domain measures, but in the diabetes group the frequency domain parameters (LF and log LF/HF were positive and HF negative) were also significant (Figure 3 and Figure 4).

### 3.5. Multiple Regression Analysis: Supine and Active Standing

Multiple linear regression analysis for supine and active standing (Table 4) confirmed that the changes in all HRV indices are associated with meanNN. The other independent factors were age (SDNN and RMSSD), diabetes mellitus condition (pNN20) or the active standing maneuver (LF, HF, and log LF/HF).

### 3.6. Multiple Regression Analysis: Supine and Rhythmic Breathing

In the supine and rhythmic breathing linear regression analysis (Table 5), meanNN and maneuver are positive predictors for time-domain parameters (SDNN, RMSSD, and pNN20); age only predicts SDNN and RMSSD, diabetes condition also predicts pNN20. The frequency-domain parameters are predicted by age and maneuver (LF and log LF/HF positive predictors, HF negative predictors).

BMI is a variable related to the metabolic condition. We tested the effect of metabolism on multivariate regression models by including BMI as an additional independent variable (Supplementary Material, Tables S1 and S2). All regression models remain similar except for SDNN and pNN20 in the combination of supine position and active standing, where the BMI was included as an independent predictor with a positive slope.

## 4. Discussion

This study tested physiological maneuvers to stimulate hemodynamic compensatory mechanisms (active standing) and the cardio-respiratory coupling (rhythmic breathing) in diabetic and healthy women, to assess the association between meanNN and the HRV indices during these controlled conditions. Our results showed that meanNN, age, and the maneuver are the main predictors of most HRV indices, while the diabetic condition was a predictor only for pNN20 during both maneuvers. Given the known reduced HRV in patients with T2DM, it is clinically important that much of the HRV indices are dependent on heart rate irrespective of the presence of T2DM. Moreover, the regression analyses evidenced the multifactorial etiology of HRV.

There were few anthropometric differences between the evaluated groups. Age, BMI, and resting HR were higher in the diabetes group. Diabetic participants were older because the age at the diagnosis is around 58 years (57.8 ± 8.7) [34], and we tried to find the population with less than 5 years of disease evolution. In our study design, the strict selection criteria for healthy participants precluded the inclusion of older participants because comorbidities are more likely at older age. Higher BMI and resting HR are related to the pathophysiology of this disease [35,36]. BMI is an important risk factor for diabetes because an excess of adipose tissue is related to insulin resistance and low-grade inflammation causing hyperglycemia [37]. However, the BMI was not an independent predictor of most HRV indices in the present study except for SDNN and pNN20 when combining data from supine position and active standing. The elevated resting HR in diabetes has been related to impaired autonomic activity due to increased sympathetic tone related to overweight and neuronal parasympathetic damage [38]. 

Regarding the metabolic profile, the glucose and uric acid blood levels were also higher in the diabetic group. Nevertheless, the diabetic participants are considered as having glycemic control and a normal renal and hepatic function [39,40]. High uric acid blood level is a factor which increases the risk of diabetes by 35% to 48% compared with non-hyperuricemic populations [41]. Some evidence indicates a bidirectional interaction between the presence of high uric acid and insulin resistance [42] which conditions long-term hyperglycemia. 

Heart rate variability (HRV) is a sensitive, non-invasive, and useful biomarker to evaluate autonomic function [43] which has been used to diagnose CAN in type 2 diabetes patients [21,22,23,24,25,26]. HRV is measured through time and frequency indices. Each is related to the activity of the autonomic nervous system, and higher variability is related to preserved autonomic activity of both sympathetic and parasympathetic branches [33]. The most used temporal parameters are SDNN, which reflects the total power of spectral analysis and the cyclic components of HRV; short term components are related to RMSSD and pNN20 [44]. Some physiological reports describe a close relationship between the activation of the sympathetic branch to LF, and parasympathetic discharge to HF and due to this, an LF/HF ratio was created to evaluate the relation between both subsystems [33].

In our study, we observed that HRV indices were lower (less variability) in the diabetes group than the healthy group in supine position and rhythmic breathing, indicating an autonomic disturbance in the normal parasympathetic resting tone and cardiorespiratory coupling. This coincides with previous findings [24,45]. In healthy subjects, the active standing maneuver increased sympathetic activity, causing an increment of LF and LF/HF indices, but in the diabetes group HF predominated, which could reflect inadequate sympathetic activation [46]. Nevertheless, given the results of the present work about the ubiquitous influence of the heart rate upon HRV indices, caution must be taken when interpreting the HRV indices in comparisons between groups from different populations (e.g., diabetic vs healthy, renal failure vs healthy, young vs old), since a baseline decreased variability in one group cannot be attributed solely to the pathology or comparison factor. Moreover, future studies are required to assess the potential influence of baseline differences in heart rate upon the response of HRV indices to a maneuver such as active standing and rhythmic breathing. Our approach was similar.

Even though the autonomic relationship with HRV has been demonstrated, some studies suggest that HRV is strongly related to average HR independent of other external stimuli [15]. We tested this hypothesis in our sample with Pearson’s correlation analysis and linear stepwise multiple regression analysis. In the healthy group, when supine and active standing data were combined to introduce a shift in the set point of HR while increasing its dynamic range, we found that meanNN maintained a significant correlation (*p* < 0.01) with all the time and frequency-domain indices. Meanwhile, in the diabetes group the significant and positive correlations appeared only in RMSSD and pNN20. This apparent loss of HR and HRV correlation in the presence of diabetic disease has not been described and could reflect that in diabetic women, the dynamic behavior of HRV loses adaptability to the orthostatic challenge [47]. In supine and rhythmic breathing, the results of the healthy group show a correlation between HR and HRV only in time-domain, because rhythmic breathing has no effect on mean HR (Table 3), and it influences the HRV indices which reflect statistical variability (time-domain HRV indices). In contrast, rhythmic breathing in the diabetic women decreased mean HR which influenced all the HRV indices except for LF/HF ratio. Rhythmic breathing, also known as paced breathing, at six breaths per minute (0.1 Hz) tends to cluster the heartbeats in inspiration, modifying the heart rate behavior in a phenomenon known as cardiorespiratory coupling [48]. At 0.1 Hz, the breathing rate increases HRV oscillations at the LF band and the baroreflex sensitivity [49]. The significant correlation of meanNN and HRV indices in the diabetes group and only in the breathing maneuver had not been reported and reflects a conserved cardiorespiratory coupling, which is lost with the progression of diabetes [24]. To date, there is no study that allows us to elucidate the reason for the increase in the correlation between HRV indices in rhythmic respiration in T2DM. The evidence supports a decrease in parasympathetic activity and baroreflex sensitivity which is expressed in a cardiorespiratory uncoupling [50] opposite to our findings. 

In our work, meanNN is the best and significant predictor of the behavior of HRV indices in supine and active standing, and only in time-domain parameters in rhythmic breathing. This demonstrates the definitive and direct relationship that exists between HRV and meanNN as reported in [15]. However, the maneuver, age, and the presence of diabetes results in independent and significant variables to predict HRV, and they have been also found in other studies in the field. Supine, active standing, and rhythmic breathing are well-known maneuvers proposed by Ewing et al., 1985 [51], that activate fast and consistent autonomic responses in healthy subjects [52], and they are widely used in clinical testing of autonomic function in metabolic [53,54], cardiovascular [55,56], psychological [57], and rheumatic diseases [58]. We expected a significant correlation with HRV indices, demonstrating the existence of cardiac autonomic activity. Normal aging is associated with a decline in cardiovascular system elasticity and compliance, and a diminished autonomic modulation [59]. These effects have been evaluated in baroreflex sensitivity and HRV indices, where both tend to decrease with age and reflect lesser parasympathetic regulation [60,61]. The sustained hyperglycemia in type 2 diabetes promotes the damage of the small amyelinic type C-fibers of the autonomic nervous systems via oxidative and nitrosative stress, which generates DNA damage, proinflammatory substances release (such as interleukin-1, tumor necrosis factor alpha, and transforming growth factor beta) and a pro-vasoconstriction profile (high levels of endothelin-1 and vascular endothelial growth factor, and a decrease in endothelial nitric oxide synthase production), which results in neuronal toxicity, histological changes, and glial death [62]. In this study, the diabetes mellitus condition results in an independent significant variable only for pNN20, which could be due to the good glycemic control of the sample with diabetes, the short duration of the disease conditions (less than 5 years of progression) and because our group does not present hyperglycemic crisis.

### Study Limitations

Our groups are not age-matched and age is an important predictor for many of the HRV indices showed in the multiple regression analysis. It was not possible for us to recruit healthy participants of the same age as the participants with diabetes because most of the diabetes participants were over 50 years. In Mexico at this age, most women start to develop obesity, dyslipidemia, hypertension, or other diseases and they must take medication for these alterations. Some of these medications, even hormonal replacement therapy as a treatment for menopause, were exclusion criteria for our work. 

For this study, we only have a fasting blood glucose level from healthy women, which confirms short-term control; an HbA1c value would be adequate to verify long-term glycemic control. 

Women with T2DM take metformin or maintain glycemic control with diet and exercise alone, allowing a more homogeneous group, but we did not perform the linear regression analysis to evaluate the correlation that could exist with metformin; for that it is necessary to recruit a larger sample.

The number of participants recruited in the present study was similar to several relevant previous studies [14,63,64]. Our sample is relatively small, due to the very strict inclusion and exclusion criteria, which may restrict the generalization of our findings but on the other hand may improve the internal validity of the results.

We did not evaluate the influence of sex-hormones fluctuations related to the sexual female cycle or menopause, which could affect the HRV indices.

In the present study, data from different conditions within the subjects (i.e., supine position, active standing, and rhythmic breathing) were combined. This approach entails a risk of bias for overestimation in the correlation analyses, since combining the samples increases the number of non-independent data points. Nevertheless, the combination of data from the resting position and autonomic challenge maneuvers aims to move the set point (meanNN) during the maneuver, which increases the dynamic range of the HRV analysis, as reported in other publications [63,65,66].

The present work evaluated the relationship between mean heart rate and HRV indices of women with T2DM and showed differences between diabetic women and healthy women in the response to a hemodynamic and a cardiorespiratory maneuver. Further studies are required to verify the generalization of our findings in men with T2DM, as well as diabetic patients (both women and men) with other relevant clinical conditions such as lack of metabolic control, other comorbidities including hypertension, the use of some medications that impact the HR and HRV such as beta-blockers, and a greater duration of the diabetes progression to show if our results are consistent.

## 5. Conclusions

This work shows clear differences of HRV indices from T2DM women with good metabolic control when compared to healthy women during supine position, with slightly faster heart rate and lower variability of heart rate, but similar sympatho-vagal balance. In response to active standing, T2DM women reached a lower sympatho-vagal balance, either by a smaller sympathetic activation or not enough parasympathetic withdrawal. Even though T2DM women reached cardiorespiratory coupling with the rhythmic breathing maneuver, they kept a lower variability of heart rate compared to healthy women.

Bivariate correlations analysis between meanNN and HRV indices showed less significant correlations in the diabetes group during active standing, and more significant correlations during rhythmic breathing. This suggests decreased adaptability of the HRV dynamics in diabetic women.

Furthermore, linear regression models demonstrated that meanNN is an independent predictor of most HRV indices, indicating that interpretation of HRV analysis in diabetic women should always consider the contribution of mean heart rate.

## Figures and Tables

**Table 1:** Anthropometric characteristics of the study participants.

Variable	Healthy Group (*N* = 44)	Diabetes Group (*N* = 19)	*p*-Value
Age (years)	42 ± 7	52 ± 7	<0.001
Height (m)	1.60 ± 0.07	1.55 ± 0.06	0.004
Weight (kg)	63 ± 7	63 ± 7	0.982
BMI (kg/m^2^)	25 ± 3	26 ± 2	0.018
Waist (cm)	86 ± 13	86 ± 7	0.817
Systolic BP (mmHg)	116 ± 13	119 ± 15	0.515
Diastolic BP (mmHg)	74 ± 9	73 ± 6	0.866
Heart rate (bpm)	61 ± 9	69 ± 7	<0.001

Data are shown as mean ± standard deviation. BMI = body mass index; BP = blood pressure.

**Table 2:** Metabolic profile variables.

Variable	Healthy Group (*N* = 44)	Diabetes Group (*N* = 19)	*p*-Value
Glucose (mg/dL)	79.86 ± 19.26	99.87 ± 20.09	<0.001
Total cholesterol (mg/dL)	163.64 ± 40.40	166.96 ± 37.33	0.730
HDL (mg/dL)	45.74 ± 15.14	48.82 ± 11.94	0.380
LDL (mg/dL)	101.47 ± 33.47	105.75 ± 32.63	0.596
Triglycerides (mg/dL)	105.94 ± 54.39	113.30 ± 29.68	0.420
Creatinine (mg/dL)	0.62 ± 0.17	0.66 ± 0.15	0.327
Uric acid (mg/dL)	3.82 ± 0.96	4.66 ± 1.37	0.034
Ureic nitrogen (mg/dL)	11.51 ± 2.73	13.29 ± 4.66	0.116

Data is shown as mean ± standard deviation.

**Table 3:** Comparison of HRV indices between groups (healthy and diabetes, *p* value in the right column) and between maneuvers (supine, active standing, and rhythmic breathing) in the same group (a, b and c markers represent *p*-value).

Variable	Healthy Group (*N* = 44)	Diabetes Group (*N* = 19)	*p*-Value
**Supine position**			
MeanNN (s)	0.990 ± 0.135 ^a^	0.920 ± 0.129 ^a,b^	0.018
SDNN (s)	0.054 ± 0.027 ^a,b^	0.041 ± 0.017 ^b^	0.022
RMSSD (ms)	47.289 ± 32.713 ^a,b^	26.722 ± 12.164 ^b^	0.001
pNN20 (%)	37.277 ± 17.440 ^a^	21.666 ± 8.605 ^a^	<0.001
LF (n.u.)	57.225 ± 18.837 ^a,b^	63.060 ± 14.685 ^b^	0.231
HF (n.u.)	43.019 ± 18.641 ^a,b^	36.940 ± 14.685 ^b^	0.203
log (LF/HF)	0.151 ± 0.379 ^a,b^	0.257 ± 0.291 ^b^	0.310
**Active standing**			
MeanNN (s)	0.811 ± 0.104 ^c^	0.822 ± 0.104 ^c^	0.714
SDNN (s)	0.047 ± 0.015 ^c^	0.037 ± 0.012 ^c^	0.008
RMSSD (ms)	24.354 ± 11.148 ^c^	19.371 ± 7.536 ^c^	0.052
pNN20 (%)	23.765 ± 14.395 ^c^	15.378 ± 9.040 ^c^	0.139
LF (n.u.)	77.512 ± 13.298 ^c^	70.153 ± 15.681 ^c^	0.034
HF (n.u.)	22.830 ± 13.256 ^c^	30.445 ± 15.858 ^c^	0.031
log (LF/HF)	0.612 ± 0.375 ^c^	0.418 ± 0.363 ^c^	0.037
**Rhythmic breathing**			
MeanNN (s)	1.025 ± 0.126	1.000 ± 0.149	0.453
SDNN (s)	0.098 ± 0.034	0.068 ± 0.025	<0.001
RMSSD (ms)	72.190 ± 32.088	49.767 ± 24.268	0.004
pNN20 (%)	32.075 ± 5.832	25.215 ± 7.544	<0.001
LF (n.u.)	89.443 ± 5.160	87.828 ± 7.107	0.273
HF (n.u.)	10.591 ± 5.137	12.223 ± 7.112	0.267
log (LF/HF)	0.979 ± 0.250	0.913 ± 0.261	0.302

Data are shown as mean ± standard deviation. ^a^ *p*-value < 0.05 supine position vs. active standing (within the same group). ^b^ *p*-value < 0.05 supine position vs. rhythmic breathing (within the same group). ^c^ *p*-value < 0.05 active standing vs. rhythmic breathing (within the same group).

**Table 4:** Linear stepwise multiple regression analysis with predicted HRV indices and, as independent variables, the meanNN, maneuver, diabetes mellitus (DM) condition, and age. The regression models were applied on the combined samples from supine position and active standing in both groups (healthy and diabetes).

Variables	Standardized β	β (C.I._95%_)	*p*-Value	R^2^
Predicted HRV index: SDNN (ms)				0.286
meanNN	0.356	52.123 (34.20–70.06)	<0.001	
Age	−0.374	−0.948 (−1.26 to −0.64)	<0.001	
Maneuver	Excluded variable			
DM condition	Excluded variable			
Predicted HRV index: RMSSD (ms)				0.492
meanNN	0.585	99.558 (81.98–117.14)	<0.001	
Age	−0.339	−1.000 (−1.31 to −0.70)	<0.001	
Maneuver	Excluded variable			
DM condition	Excluded variable			
Predicted HRV index: pNN20 (%)				0.349
meanNN	0.510	58.317 (44.94–71.69)	<0.001	
DM condition	−0.268	−10.289 (−14.78 to −5.80)	<0.001	
Maneuver	Excluded variable			
Age	Excluded variable			
Predicted HRV index: LF (n.u.)				0.253
meanNN	−0.248	−31.338 (−50.27 to −12.41)	0.001	
Maneuver	0.331	12.064 (6.60–17.52)	<0.001	
DM condition	Excluded variable			
Age	Excluded variable			
Predicted HRV index: HF (n.u.)				0.248
meanNN	0.241	30.320 (11.43–49.21)	0.002	
Maneuver	−0.331	−12.006(−17.46 to −6.56)	<0.001	
DM condition	Excluded variable			
Age	Excluded variable			
Predicted HRV index: log (LF/HF)				0.249
meanNN	−0.239	-0.691 (−1.12 to −0.26)	0.002	
Maneuver	0.334	0.278 (0.153–0.403)	<0.001	
DM condition	Excluded variable			
Age	Excluded variable			

**Table 5:** Linear stepwise multiple regression analysis with predicted HRV indices and, as independent variables, the meanNN, maneuver, diabetes mellitus (DM) condition, and age. The regression models were applied on the combined samples from supine position and rhythmic breathing in both groups (healthy and diabetes).

Variables	Standardized β	β (C.I._95%_)	*p*-Value	R^2^
Predicted HRV index: SDNN (ms)				0.526
meanNN	0.241	62.414 (33.56–91.27)	<0.001	
Age	−0.369	−1.525 (−1.98 to −1.07)	<0.001	
Maneuver	0.492	17.451 (13.67–21.23)	<0.001	
DM condition	Excluded variable			
Predicted HRV index: RMSSD (ms)				0.510
meanNN	0.351	84.576 (57.31–111.84)	<0.001	
Age	−0.436	−1.671 (−2.10 to −1.24)	<0.001	
Maneuver	0.282	9.282 (5.71–12.86)	<0.001	
DM condition	Excluded variable			
Predicted HRV index: pNN20 (%)				0.265
meanNN	0.361	36.337 (22.82–49.86)	<0.001	
DM condition	−0.316	−9.675 (−13.75 to −5.60)	<0.001	
Maneuver	−0.163	−2.246 (−4.08 to −0.41)	0.017	
Age	Excluded variable			
Predicted HRV index: LF (n.u.)				0.551
Age	0.135	0.325 (0.08–0.57)	0.010	
Maneuver	0.736	15.206 (13.09–17.32)	<0.001	
meanNN	Excluded variable			
DM condition	Excluded variable			
Predicted HRV index: HF (n.u.)				0.557
Age	−0.135	−0.324 (−0.57–−0.08)	0.010	
Maneuver	−0.740	−15.278 (−17.38 to −13.18)	<0.001	
meanNN	Excluded variable			
DM condition	Excluded variable			
Predicted HRV index: log (LF/HF)				0.612
Age	0.124	0.007 (0.002–0.013)	0.011	
Maneuver	0.778	0.392 (0.34–0.44)	<0.001	
meanNN	Excluded variable			
DM condition	Excluded variable			

## Data Availability

The data presented in this study are available on request from the corresponding author.

## Supplementary Materials

The following are available online at https://www.mdpi.com/article/10.3390/jcm10194386/s1, Table S1: Linear stepwise multiple regression analysis with predicted HRV indices and as independent variables the meanNN, maneuver, diabetes mellitus (DM) condition, age, and BMI. The regression models were applied on the combined samples from supine position and active standing, Table S2: Linear stepwise multiple regression analysis with predicted HRV indices and as independent variables the meanNN, maneuver, diabetes mellitus (DM) condition, age, BMI, glucose, and uric acid. The regression models were applied on the combined samples from supine position and rhythmic breathing,
