# Supplementary material for: Dependence of Heart Rate Variability Indices on the Mean Heart Rate in Women with Well-Controlled Type 2 Diabetes
Source: J Clin Med. 2021 Sep 25;10(19):4386. doi: 10.3390/jcm10194386 (PMC8509544; doi:10.3390/jcm10194386)
Supplement: Supplementary file 1 [file jcm-10-04386-s001.zip › jcm-1306334-supplementary.pdf]

Supplementary material

Table S1. Linear stepwise multiple regression analysis with predicted HRV indices and as independent variables the meanNN, maneuver, diabetes mellitus (DM) condition, age, and BMI. The regression models were applied on the combined samples from supine position and active standing.

Variables	Standardized β	β (C.I.$_{95\%}$)	P	R^2
Predicted HRV index: SDNN (ms)				0.322
meanNN	0.342	49.711 (32.057 – 67.366)	<0.001	
Age	-0.427	-1.074 (-1.39 – -0.76)	<0.001	
BMI	0.186	1.520 (0.498– 2.54)	0.004	
DM condition		*Excluded variable*		
Maneuver		*Excluded variable*		
Predicted HRV index: RMSSD (ms)				0.495
meanNN	0.586	99.895 (82.13–117.66)	<0.001	
Age	-0.342	-1.008 (-1.32– -0.70)	<0.001	
Maneuver		*Excluded variable*		
DM condition		*Excluded variable*		
BMI		*Excluded variable*		
Predicted HRV index: pNN20 (%)				0.393
meanNN	0.493	55.597 (42.66 – 68.54)	< 0.001	
DM condition	-0.320	-12.09 (-16.55 – -7.63)	<0.001	
BMI	0.215	1.362(0.61 – 2.11)	<0.001	
Age		*Excluded variable*		
Maneuver		*Excluded variable*		
Predicted HRV index: LF (n.u.)				0.254
meanNN	-0.246	-31.060 (-50.09 – -12.03)	0.002	
Maneuver	0.334	12.237 (6.71 – 17.76)	<0.001	
Age		*Excluded variable*		
DM condition		*Excluded variable*		
BMI		*Excluded variable*		
Predicted HRV index: HF (n.u.)				0.248
meanNN	0.239	30.043 (11.05 – 49.04)	0.002	
Maneuver	-0.334	-12.174 (-17.69 – -6.66)	<0.001	
Age		*Excluded variable*		
DM condition		*Excluded variable*		
BMI		*Excluded variable*		
Predicted HRV index: log (LF/HF)				0.250
meanNN	-0.238	-0.686 (-1.12 – -0.25)	0.002	
Maneuver	0.338	0.283 (0.156 – 0.409)	<0.001	
Age		*Excluded variable*		
DM condition		*Excluded variable*		
BMI		*Excluded variable*		

Table S2. Linear stepwise multiple regression analysis with predicted HRV indices and as independent variables the meanNN, maneuver, diabetes mellitus (DM) condition, age, BMI, glucose, and uric acid. The regression models were applied on the combined samples from supine position and rhythmic breathing.

Variables	Standardized β	β (C.I.95%)	P	R^2
Predicted HRV index: SDNN (ms)				0.530
meanNN	0.242	62.477 (33.71 – 91.25)	<0.001	
Age	-0.373	-1.533 (-1.99 – -1.08)	<0.001	
Maneuver	0.490	17.368 (13.58– 21.16)	<0.001	
DM condition		*Excluded variable*		
BMI		*Excluded variable*		
Predicted HRV index: RMSSD (ms)				0.512
meanNN	0.352	84.565 (57.24 – 111.89)	<0.001	
Age	-0.439	-1.681 (-2 .11 – -1.25)	<0.001	
Maneuver	0.276	9.118 (5.52 – 12 .71)	<0.001	
DM condition		*Excluded variable*		
BMI		*Excluded variable*		
Predicted HRV index: pNN20 (%)				0.270
meanNN	0.370	36.457 (23.16 – 49.76)	< 0.001	
DM condition	-0.316	-9.522 (-13.54 – -5.51)	<0.001	
Maneuver	-0.158	-2.147 (-3.96 – 0.33)	0.021	
Age		*Excluded variable*		
BMI		*Excluded variable*		
Predicted HRV index: LF (n.u.)				0.549
Age	0.134	0.321 (0.07 – 0.57)	0.011	
Maneuver	0.736	15.166 (13.04 – 17.30)	<0.001	
meanNN		*Excluded variable*		
DM condition		*Excluded variable*		
BMI		*Excluded variable*		
Predicted HRV index: HF (n.u.)				0.555
Age	-0.134	-0.321(-0.57 – -0.08)	0.011	
Maneuver	-0.740	-15.241 (-17.35 – -13.13)	<0.001	
meanNN		*Excluded variable*		
DM condition		*Excluded variable*		
BMI		*Excluded variable*		
Predicted HRV index: log (LF/HF)				0.609
Age	0.123	0.007 (0.002 – 0.013)	0.012	
Maneuver	0.777	0.391 (0.343 – 0.440)	<0.001	
meanNN		*Excluded variable*		
DM condition		*Excluded variable*		
BMI		*Excluded variable*		
